# Quality of antenatal care in selected public health facilities of West Ethiopia

**DOI:** 10.1186/s40834-022-00186-9

**Published:** 2022-10-01

**Authors:** Amanuel Nemomsa, Desalegn Wirtu, Motuma Getachew, Gemechu Kejela, Emiru Merdassa, Workineh Diriba, Markos Desalegn

**Affiliations:** 1 Department of Public health, Institute of health science, Wollega University, Nekemte, Ethiopia; 2 School of medicine, college of medicine and health science, Jigjiga University, Jigjiga, Ethiopia

**Keywords:** Antenatal care, Service, Quality, West Ethiopia

## Abstract

**Background:**

Ante natal care (ANC) is a key entry point for a pregnant woman to receive abroad range of promotion and preventive health services. Quality of ANC has paramount role to ensure better maternal and neonatal outcome.

**Objective:**

To assess the quality of antenatal care services at public health facilities of western Ethiopia.

**Methods:**

Facility-based cross-sectional study was conducted from May 30th to June 30th, 2016. All public health facilities in the Kellem Wollega Zone of West Ethiopia were audited, 316 medical records were reviewed, and 316 pregnant women were interviewed. The data was entered using EPI Data version 3.1 and analyzed using SPSS version 20.0. Descriptive statistics, binary, and multiple logistic regressions were conducted. Variables with a *P*-value of <0.05 were considered as statistically significant associated factors.

**Results:**

A total of 316 pregnant women were enrolled in the study. All facilities were categorized as “good” by the possession of necessary equipment, 3/4 by basic amenities and 87.34% by general and gynecologic examination. The information was provided for 222(86.21%), which is categorized as poor. About 252 (79.7%) of the women were satisfied with ANC. A urine sample taken during ANC visit [(AOR= 3.36 (95 % CI= 1.70, 6.61)], and counseling on nutrition during pregnancy [(AOR= 2.27 (95 % CI=1.16, 4.45)] were predictors of client satisfaction on ANC.

**Conclusions:**

In this study quality of ANC was labeled good for structural aspects and poor for process aspects of quality. In terms of outcome aspects, the majority of pregnant women were satisfied with the ANC they received. A urine sample taken during the ANC visits and being counseled on nutrition during pregnancy were predictors for client satisfaction on ANC. Concerned bodies need to improve laboratory tests and information provision.

## Background

According to the World Health Organization (WHO), Antenatal care (ANC) is the care that a woman receives during pregnancy, that helps to ensure healthy outcomes for women and newborns. It is a key entry point for a pregnant woman to receive abroad range of promotion and preventive health services. To achieve its objectives, providing quality ANC for pregnant women is crucial. However, as evidences indicate, the quality of ANC is poor in almost all developing countries [1].

In Ethiopia, as in most developing countries, quality improvement efforts are slow and scattered. In the country, only 41% of women received antenatal care once in their course of pregnancy and one in every four women reported that they were informed of signs of pregnancy complications during their ANC visit, 67 % had a blood sample taken, 52 % had a urine sample taken, 71% had their blood pressure measured and only 34 % took the iron tablet and only 16% of pregnant women gave birth by skilled providers [2]. In Ethiopia, pregnant mothers attending ANC clinics were found to receive only part of recommended care components and almost half (47.7%) of the study women were not satisfied and a large proportion of mothers are missing opportunities to receive screening (like blood pressure and weight measurements) and preventive components of antenatal care (iron/folic acid supplementation) [3].

The new approach to ANC emphasizes the quality of care rather than quantity [4]. However, Evidences suggested that, not more than one third of the pregnant women receive a good quality ANC service [5–7]. Studies have shown that there are many missed opportunities for care, both because of the client- and health system-related factors. Mothers and children may face risks because of limited or close to term ANC visits, low-quality care during visits due to poor provider training, infrastructure and administrative weakness at facilities, complications of existing conditions such as TB, malaria, anemia, or sexually transmitted infections (STIs), and short intervals between births [8].

Even though the quality of ANC practice is one of the important strategies to reduce maternal and newborn morbidity and mortality, the study conducted to assess the quality of ANC practice in Ethiopia is limited in number. The frequency of ANC visit alone is not an end to save the lives of the mothers and newborn unless the mothers can receive the quality ANC to the level of the standards.

Therefore, this study assessed the level of quality of ANC in public health facilities of Kellem Wollega Zone. The result of the study can be used to increase the client satisfaction with ANC services from public health facilities.

## Methods

### Study design, area and period

A facility-based cross-sectional study was conducted in public health facilities of Kellem Wollega Zone, western Ethiopia from May 30 to June 30, 2016. Kellem Wollega zone is one of the eighteen zones of Oromia Regional State. Based on the 2007 Census conducted by the Central Statistical Agency of Ethiopia (CSA), this Zone has a total population of 797,666, of which 401,905 are men and 395,761 are women. By residence, 76,277 (9.56%) of the population are urban inhabitants. A total of 159,353 households were counted in this Zone, which results in an average of 5.01 persons per household [9]. According to the 2015/2016 district based plan, there are about 34,470 of women expected to be pregnant in the year in the zone by conversion factors. Forty-five Health Centers and one zonal and one district hospital are currently providing ANC services in this zone [10].

### Population and eligibility criteria

All pregnant women, who were attending ANC clinic, and all health facilities in the Kellem Wollega zone, were the source population. Selected health facilities, pregnant women attending ANC clinic at the selected health facilities in Kellem Wollega Zone were the study population. Pregnant women who were seriously ill, with a mental health problem and hearing impairment were excluded from the study.

### Sample size determination

Sample size was determined using single population proportion formula, by considering the proportion of satisfied pregnant women from study done in Ambo town which was 89% [11], marginal of error of 5%, and confidence level of 95%.

After multiplying the calculated sample size by 2 for design effect and adding 5% for non-response rate, the final sample size becomes 316 pregnant mothers.

### Sampling procedure

There are ten districts in the Kellem Wollega zone. Seven of them were selected by a simple random sampling technique using the lottery method. One health center was selected from each district and the lottery method was used when more than one facility exists in the district. Dembi Dollo Hospital was selected from the two public hospitals in the zone. The total calculated sample size was proportionally allocated to each health facility according to the number of attendants of the recent quarter ANC report. Finally, pregnant women who were registered and attending the antenatal care service during the data collection period were taken consecutively until the required sample size from the specific facility was fulfilled.

### Data collection tools and procedures

A structured questionnaire was adapted from the Safe Motherhood Needs Assessment package to obtain information on services received by pregnant women during exit interviews [12]. Satisfaction level was measured by 12 items of questions prepared to measure the level of satisfaction, adopted from similar studies conducted in the country. Satisfaction level with a Likert scale of 1 to 5 score and the mean satisfaction score was used as a cut-off point.

A checklist for client medical review was adopted as per the Ethiopian Federal Ministry of Health integrated cards of ANC, labor, delivery, newborn, and post-natal care.

Eight clinical nurses and two MPH holder supervisors were selected from facilities that were not included in the study. Two days of training were given for data collectors and supervisors on data collection techniques and all data collection tools. The data collectors conducted client exit interview and review of medical records.

#### Client exit interview

Clients were interviewed after they finished the whole process of antenatal care services at the exit area.

Data collectors reviewed all respondents’ medical records including the types of services clients received during their ANC visit on daily basis for all interviewed pregnant women. The attributes of output and some parts of process components of ANC quality were assessed from the client interview.

#### Facility audit

All selected facilities were audited to assess the health facilities’ capacity to deliver ANC services.

The components for structural aspects of ANC quality (Management system, Basic amenities, Infection prevention materials, ANC Diagnostic equipment, Laboratory equipment, and Essential drugs) were obtained from the audit.

#### Medical record review

A medical recording number was written on individual questionnaires to differentiate client medical records. Medical record review was done using a checklist adopted as per the Ethiopian Federal Ministry of Health integrated cards of ANC, labor, delivery, newborn and postnatal care service. The most components for process aspects of ANC quality (General, gynecological examination, and routine ANC tests done, drugs, and information given for pregnant women) were assessed.

### Operational definitions

#### Quality of ANC services

Based on Donabedian’s quality of care framework, this study assessed three aspects of the quality of care: structure, process, and outcome.

#### Structural aspects of quality

To assess structural aspects of the quality, six different components (Management system, Basic amenities, Diagnostic equipment, Infection prevention materials, Laboratory equipment, and Essential drugs) were used and indices for each of them were generated. To generate an index, each variable was used to assess the six different components of structural aspects that were scored, and then the sum of the scores was obtained. For example, the management system index had three variables in which each was given a score of ‘1’ if present and ‘0’ if absent. The management system index, therefore, had values ranging from (0-3). A similar approach was used for the generation of indices for Basic amenities, Diagnostic equipment, Infection prevention materials, Laboratory equipment, and Essential drugs. The Basic amenities index had values ranging from (0-7), Diagnostic equipment index had values ranging from (0-8), Infection prevention materials index had values ranging from (0-8), Laboratory equipment index had values ranging from (0-6), and Essential drugs index had values ranging from (0-4), depending on the number of variables assessed in each of them as described under the operational definition above.

To compute comparison between the health facilities’ structural aspects of the quality, the indices of basic amenities, diagnostic equipment, infection prevention materials, and laboratory equipment were categorized into two,” Poor or good”.

The structural aspects of the quality were graded as poor if its categorized index value was between zero and four and as good if the value was above five [13]. While for the Management system and Essential drugs, the indices generated were not categorized and the results presented in narrative and table.

#### Process aspects of quality

To assess process aspects of the quality, four different components (General and Gynecological examination are done, Routine ANC tests are done, Drugs are given and Information provided for pregnant women) were used and indices for each of them were also generated. The variables described under each of these categories or components were first scored, such that a variable was given a score of one if undertaken and zero if not undertaken, and then the scores were summed under each of the components described above to generate an index. General and Gynecological examination done index had values ranging from (0-12), Routine ANC tests are done index had values ranging from (0-6), Drugs given index had values ranging from (0-3), and Information provided for pregnant women index had values ranging from (0-8). To compute comparison between the health facilities process aspects of the quality, the indices of General and Gynecological examination were categorized, and Information provided for pregnant women was categorized into two categories,” Poor or good”. The process aspects of the quality were graded as ‘poor’ if its index value was between zero and six, and ‘good’ if the value was seven and above [13]. While for Routine ANC tests done and Drugs are given, the indices generated were not categorized and the results presented by the bar graph and narrated.

#### Outcome aspects of quality

Pregnant women’s satisfaction was used to assess the outcome aspects of the quality of ANC services. Pregnant women's satisfaction was measured by statements related to health services prepared to measure the satisfaction of clients. Clients were categorized as satisfied (if they score above the mean satisfaction score) or not satisfied (if they score equals or below the mean) by using Client satisfaction that was rated by 12 items of health service statements.

These statements were related to satisfaction of client on provider behavior, communication skill, waiting time, Clinic amenities, waiting area, laboratory tests, and drugs are given, privacy during the consultation, procedure, information received about ANC services, continuity of care, the recommendation to other services and a general feeling of the client. Each question has one to five-point of Likert scales.

### Data quality assurance

The questionnaire was prepared in English. Then it was translated to Afaan Oromo and translated back to English by language professionals for its consistency. Two days of training was given for data collectors and supervisors. The Questionnaire was pretested on 15 pregnant women out of the study area. Medical records were standard tool as it was developed nationally and providers have training on health information management system. During data collection time, the data collectors were closely supervised. The questionnaire and checklist were checked for completeness and consistency.

### Data processing and analysis

Following the data collection, data were coded and, entered into EPI Data version 3.1 and exported to SPSS version 20.0 for analysis.

Descriptive statistics and logistic regression analysis were performed for client satisfaction. In the logistic regression, both Bivariate and multivariate analyses were carried out to identify factors associated with client satisfaction. Assumptions of logistic regression like model fitness, multicolinearity and normality were checked. All the variables were entered into Bivariate logistic regression analysis and those explanatory variables with a *p* value<0.25 in the crude analysis were considered as a candidate for multivariate logistic regression analysis and those variables with a *p*-value < 0.05 in the multivariate logistic regression analysis were considered as a significant predictors of satisfaction. Finally, the results of the analysis were reported in texts, tables, and graphs as appropriate.

## Results

### Socio demographic characteristics of the respondents

A total of 316 pregnant women were enrolled in the study making the response rate of 100%. The majority 130(41.13%) of pregnant women belongs to the age range of 15-19 years. Three hundred seven (97.2%) of the women were married, 6(1.9%) single and the rest three (0.9%) were divorced. The predominant ethnic group of the respondents was Oromo 306(96.8%) and 10(3.2%) were Amhara (Table 1).

**Table 1:** Socio demographic factors of the pregnant women attending ANC clinic at public health facilities in Kellem Wollega zone, June 2016

Socio demographic factors	Frequency	Percentage
**Age category of the respondents in Years**		
15-19	130	41.13
20-24	109	34.5
25-29	48	15.2
30-34	28	8.9
**Occupation of the respondents**		
House wife	226	71.5
Government Employee	29	9.2
Student	20	6.3
Merchants	17	5.4
Daily laborer	9	2.8
Local alcohol seller	6	1.8
Others^a^	9	2.8
**Educational level of the respondents**		
Cannot write and read	66	20.9
Primary (1-8 Grade)	137	43.4
Secondary school (9-10 Grade)	66	20.9
Preparatory school (11-12 Grade)	10	3.2
College and above	37	11.7
**Religion of the respondents**		
Protestant	181	57.3
Muslim	81	25.6
Orthodox	39	12.3
Others^b^	15	4.7

Others^a^= Jobless, self-employee

Others^b^= Wakefata, Adventist

### Obstetric and reproductive health characteristics

Among the total study participants, 195(61.70%) had given birth to one to four children and Concerning the number of antenatal care visits for the current pregnancy, 113 (35.8%) visited the ANC clinic for the second time flowed by 98(31%)first, 77(24.4) third and 28(8.9%) fourth (Table 2).

**Table 2:** Reproductive profile of the pregnant women attending ANC clinic at public health facilities at Kellem Wollega zone, 2016

S.No	**Reproductive profile of the pregnant women (*N*=316)**		
	Parity	Frequency	Percent
1.	Null Para	95	30.1
2.	Multi-Para	195	61.7
3.	Grand Multi-Para	26	8.2
	**Obstetric profile of the pregnant women (*N*=221)**		
1.	Live birth	209	94.6
2.	Had history of Child Death	25	11.3
3.	History of Abortion	31	14.0

### Structural attributes of the quality

**Fig. 1:**
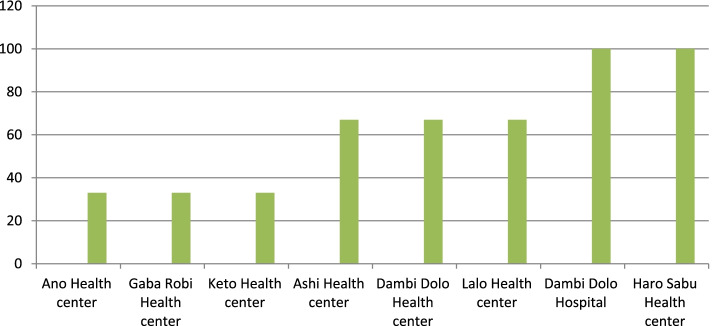
Components of management system by percentage among public health facility of Kellem Wollega Zone, West Ethiopia, June, 2016

Only Two facilities had all the components of the management system while other facilities have less than 50% of the components. At 7 of the facilities, all ANC service providers reported having taken training on ANC. All facilities had assigned midwives for ANC and delivery services and also had a minimum of two providers at the facilities. Five of the public health facilities had not supervised for their MCH activities by external bodies within the past six months (Fig. 1).

Regarding the basic amenities, 6(75%) of the facilities were categorized as good in having basic amenities. However, 3(37.5%) of the facilities had neither a private ANC room nor a private toilet.

With regards to the infection prevention materials as FMOH infection prevention guidelines, all health facilities have adequate infection prevention materials and are categorized as good. Soap for hand washing, sterilizer, waste disposal bin, sharp container, latex glove, and anti-septic solutions were seen at all health facilities during data collection. But, almost 50% of the facilities have no disinfectant solution and one facility reported that there was no water source in the compound during the data collection period.

Regarding diagnostic equipment, all the facilities were categorized as good as most of the important diagnostic equipment was seen during a facility audit. Gynecological examination table, blood pressure measuring apparatus, stethoscope, fetoscope, and vaginal speculum were seen and were functional at all facilities.

Gynecological examination light and thermometer were seen only in 2(25%) and 3(37.5%) of the facilities respectively. None of the facilities has a tape meter for uterine size measuring.

All the facilities had microscopy, urine analysis/ urine dipstick tests, blood group and RH tests, pregnancy tests, and blood film test for malaria. VDRL for syphilis test was seen at five facilities and Hemoglobin test at seven facilities. HIV test algorithm was seen at only two facilities.

All health facilities have essential drugs (Iron, Mebendazole, TT vaccine, and ART drugs) for ANC services as per the standards.

### Process attributes-interpersonal aspects

Regarding the interpersonal relationship between providers and clients, 273(86.4%) of the clients reported that the provider discusses with them anything that bothers them and 285(90.2%) of the clients said that the providers attentively listened to their concerns during their ANC checkups.

The clients were asked to rank the time they spent with providers during the consultation and ranked as about the right time by 190 (60.1%), a short time, 68(21.5%), a long time by50 (15.8%) of the respondents.

About 122(38.6%), of the clients, reported that there was another person in the ANC service room other than the provider during their consultation time that worried them, that their privacy was not maintained.

Regarding providers’ sex preferences, the majority, 144(45.6%) reported no providers’ sex preferences, but, 135(42.7%) and 37(11.7%) preferred to be seen by female and male providers respectively if they are given the chances to choose.

### Process attributes-technical aspects

In this study, the technical aspects of the process were assessed as per the recommendation to each visit and gestational age. General and gynecological examination is done, routine ANC laboratory tests done, drugs are given and information provided for pregnant women were the technical aspects assessed. The general and gynecological examination done, 276(87.34%) of the pregnant women received services and were categorized as good as per the criteria cited for this particular study.

### Basic ANC laboratory tests done and drugs given

**Fig. 2:**
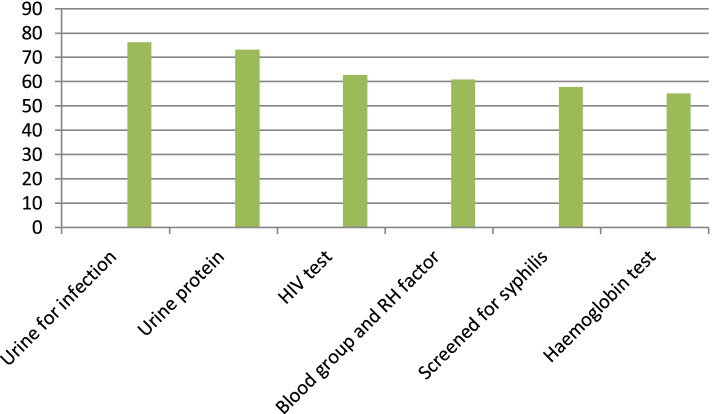
Types of laboratory test by percentage among pregnant women attending ANC in public health facility in kellem Wollega zone, west Ethiopia, June, 2016

Among all study participants asked for whether their blood and urine sample had taken, 267(84.5%) and 2235(74.4%) reported that urine and blood samples had taken respectively. The data from medical record review showed that, among all pregnant women who were given basic laboratory services, 241(76.3%) tested urine for infection, 231(73.1%) and 174(55.4%) tested urine protein and hemoglobin respectively. About 255 (80.7%) of clients reported that Iron pills were given and 281(88.9) of them taken the TT vaccine (Fig. 2).

### Information provided for pregnant women during ANC visit

**Fig. 3:**
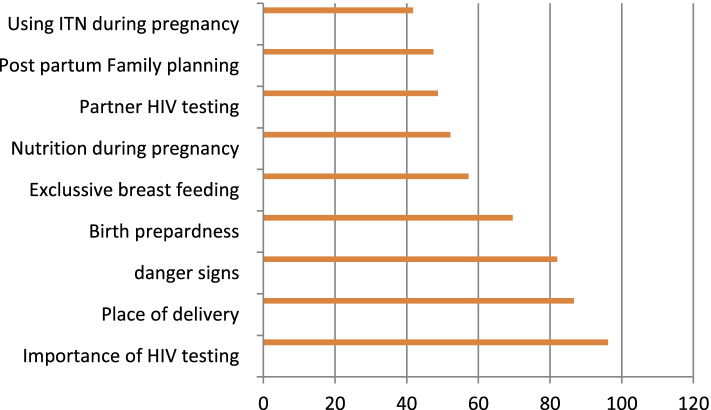
Information provided and information type during ANC among pregnant women attending ANC in public health facility in kellem Wollega zone, west Ethiopia, June, 2016

Information provided for 222(86.21%) pregnant women were categorized as poor as per the criteria cited for this particular study. The most commonly provided information was the importance of HIV testing during pregnancy 304(96.2%), followed by counseling about places of delivery 274(86.7%) and danger sign that may happen during pregnancy and delivery 259(82%). The least provided information was the importance of postpartum family planning 150(47.5%) and using of the ITN during pregnancy 132(41.8%) (Fig. 3).

### Client satisfaction

About 252 (79.7%) of the pregnant women were scored greater than the mean satisfaction score while the rest 64(20.3%) of the pregnant women scored less than the mean satisfaction score and categorized as dissatisfied. The major items or types of services in the clinics with low satisfaction scores were cleanness of latrine and adequate water supply, good laboratory tests, and medicine given.

### Predictors of client satisfaction with antenatal care

To assess the association of independent variables with the outcome variable (client satisfaction), binary and multiple logistic regression analyses was computed. In binary logistic regression analysis; blood and urine samples taken, Iron tablets given, counseling on the importance of using ITN during pregnancy, importance and types of food to be eaten during pregnancy, danger sign that might happen during pregnancy, labor, and postnatal period and birth preparedness were a candidate for multiple logistic regression.

In multiple logistic regression analyses; a urine sample taken during one of ANC visits AOR 3.36(1.70, 6.61) and counseling on importance and types of food to be eaten during pregnancy AOR 2.27(1.16, 4.45) were predictors of client satisfaction on ANC services (Table 3).

**Table 3:** Lists of variables seen to be candidate for multiple logistic regression and predictor variable for client satisfaction among ANC attending pregnant women in public health facilities of Kellem Wollega Zone, West Ethiopia, June 2016

Variables	***P***-Value	AOR	95% C.I.	
			Lower	Upper
Counseled on importance of recognizing danger symptoms that may happen during pregnancy or delivery	0.078	1.963	0.927	4.159
Blood sample taken during any of your visit	0.278	1.59	0.688	3.673
Urine sample taken during ANC Visit	0.001	3.361	1.709	6.612
Counseled on importance of using ITN during pregnancy	0.24	0.671	0.345	1.305
Counseled on birth preparedness and complication readiness	0.655	1.166	0.594	2.29
Counseled on importance of types of food to eat during pregnancy)	0.017	2.271	1.16	4.447
Iron tablets given during ANC	0.212	0.627	0.30	1.306

## Discussion

According to this study, a different attribute of quality ANC services varies from facility to facility. From structural attributes, only two facilities have all the components of the management system, while other facilities have less than 50% of the components, the majority of the facilities were categorized as good by basic amenities and infection prevention materials were available at all the assessed facilities. Regarding diagnostic equipment, all facilities were categorized as good. Laboratory equipment for ANC varies from facility to facility. Seventy-five percent of the facilities were categorized as having good laboratory equipment and all facilities had essential drugs used for ANC services.

The finding of diagnostic equipment from the current study is comparable to a study conducted in Eastern Uganda. The study also found out that the availability of physical infrastructure and diagnostic equipment was good in the health facilities however the availability of essential drugs was generally poor which is different from the finding of the current study [13]. The differences in essential drugs might be due to the current Ethiopian health care finance and integrated pharmaceutical and logistic system.

Regarding the process aspects of quality, in this study, most of the respondents reported that the providers seem interested to listen to their concern actively which is in line with the study conducted in Bahir Dar and Ambo town, where 365 (98.9%) and 206(71.5%) of respondents reported that the providers seem interested in listening to their problem respectively [3, 11].

In this study, the privacy and confidentiality of clients were not secured during consultation time.

This might be because three of the facilities in this study area had no private ANC room and they were providing the services with other services like family planning and Immunization. Also finding from Bahir Dar showed that, 51 (13.8%) of the study participants reported that there were people other than the provider during the consultation [3].

Laboratory tests done for pregnant women in this study were Rh factors 226(71.5%), syphilis tests 183(57.9%), Hemoglobin tests174 (55.1%), urine protein tests 241(76.3%), and HIV test 198(62.7%). The study conducted in Bahir Dar especial zone showed that laboratory tests done were blood group and Rh 133(36%), syphilis tests 73(31.7%), Hemoglobin 113(30.6%), urine protein 128(31.7%), and HIV test 304(82.3%) [3]. The finding of the current study was better than that of Bahir Dar almost in all laboratory tests except HIV test. The difference between the studies might be due to the current attention paid to improve maternal and child health from government and other partners, exempted maternal and child health services. The HIV test service in the current study was lower and this could be due to the HIV test kits shortage during the study period.

In this study, almost all 315(99.7%) of pregnant women’s weight had measured, 211(66.8%) of pregnant women’s gestational age were estimated, 252(82%) of them had measured their blood pressure. Another study conducted at Jimma town, which demonstrated that the blood pressure and weight of 33 (8.9%) and 17 (4.6%) of the study women were not measured, respectively [14]. The difference of finding between the studies might be due to the training status of ANC service providers’

Client’s satisfaction affects clinical outcomes and client retention. In the current study, 252(79.7%) of pregnant women were satisfied with ANC services they received from public health facilities. The finding of this study is similar with the study conducted in southwest Nigeria (81.4%) [15], and study conducted in Gambia (79.9%) [16]. the current prevalence is high compared with studies conducted in Jimma (60.4%) [14] and Bahir Dar (52.3%) [3]. On the other hand, the finding of the current study is lower than the study conducted in Ambo (89%) [11], study conducted in south east Nigeria (89.7%) [17], and study conducted in Egypt (97%) [18]. The possible reason for the difference in finding might be related to the year of study, availability of qualified health professionals like midwife nurses and ANC trained providers at the study area, modern infrastructure and good care.

A urine sample taken during one of ANC and being counseled for nutrition during pregnancy were predictors of client satisfaction in this study. This may be because clients who obtained the service they want are more satisfied than those who failed to get the service they assumed. However, these variables were not found to be associated factors with client satisfaction on ANC service in other studies [3, 11, 13, 18–21]. This finding could be very important because these variables were identified in this study as predictor of client satisfaction of ANC service so that it helps the program managers and service provider to focus on these ANC service components.

## Limitation

The study used a cross-sectional design that cannot establish trends and causality between the Quality of ANC and associated factors. Also, social desirability bias leads to under-reporting of the important component for measuring the quality of ANC. Not only this, but also lacked of appropriate literatures made us use very few number of references.

## Concussion

The findings of the current study showed that all the WHO recommendations didn’t fulfilled. The majority of the pregnant women (79.7%) were satisfied with the antenatal care services they received. A urine sample taken during one of the ANC visits, and counseled on the importance and types of food to be eaten during pregnancy were predictor variables for client satisfaction on ANC services.

## Recommendation

Privacy of clients should be secured by making ANC clinic a private room and avoiding other persons entering the procedure room other than the provider during consultation time and all ANC service providers and facilities heads are responsible.

All pregnant women have to get access to disease screening like syphilis and HIV tests as these infections to affect the health of both mothers and newborns. Facilities’ heads, district health office, zonal, and regional health bureau are responsible to avail the necessary tests and supplies in continuity.

Cleanliness of latrine, water supply, and laboratory services are things to be improved at all assessed facilities to increase the satisfaction level of pregnant women with ANC service and respective facilities’ heads are responsible.

## Data Availability

The data collected for this study can be obtained from the corresponding author based on a reasonable request.

## Abbreviations

AIDS: Acquired Immune Deficiency Syndrome
ANC: Ante Natal Care
AOR: Adjusted Odd Ratio
BP: Blood Pressure
COR: Crude Odd Ratio
EDHS: Ethiopian Demographic Health Survey
HGB: Hemoglobin
HIV: Human Immunodeficiency Virus
ITN: Insecticide Treated Nets
PMTCT: Prevention of Mother to Child Transmission
STI: Sexually Transmitted Infection
TB: Tuberculosis
VDRL: Venereal Disease Research Laboratory
WHO: World Health Organization
